# Can you keep up? The challenges for research institutes and core facilities in scouting and adopting new technologies

**DOI:** 10.1038/s44319-024-00078-w

**Published:** 2024-02-19

**Authors:** Geert Van Minnebruggen, Saskia Lippens

**Affiliations:** VIB Technologies, VIB, Flanders Institute for Biotechnology, Ghent, Belgium

**Keywords:** Economics, Law & Politics, History & Philosophy of Science, Methods & Resources

## Abstract

The rapid pace of technology evolution puts pressure on scientists, research institutes and core facilities to explore and embrace the latest developments. Cooperation and various testing strategies are key to efficiently decide on which platforms are promising and worthwhile to adopt.

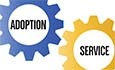

Life scientists and research institutes have to keep up with quickly evolving new technologies and methods. Only a strong collaborative approach between technology experts and researchers can make all the necessary elements within the tech cycle—from scouting to de-risking, implementation, and provision of services—come together and work together.

Indeed, the pace of technology development and evolution is dazzling. Since Silicon Valley propelled the third industrial revolution that ushered in electronics, advanced communications, automation, and space exploration, technological advancements have been transforming society on many levels. Who could have foreseen the dotcom burst after the internet’s public debut in 1991? Or the life science revolution since the release of the first draft of the human genome in 2000? When we look at how these technology-driven revolutions have affected research in the life sciences, it becomes clear that core facilities face a critical choice: evolve and adapt to new developments or become obsolete. Imagine where life scientists would stand without revolutionizing tools such as CRISPR-Cas gene-editing, synthetic biology, resolution-breaking microscopes, new single-cell and/or spatial omics techniques, and many more.

Core facilities have been supporting scientific research by acting as centralized hubs that bundle specialized equipment and experienced staff to offer services that go beyond the capabilities of individual labs. While the early facilities initially provided access to high-end research equipment in a cost-effective manner, they eventually transitioned into knowledge centers, pairing top-tier equipment with expert guidance. Today, cores are universally recognized as corner stones of research institutes with a mission to boost research. Consequently, core facilities must remain agile and constantly adapt to embrace the latest advancements in scientific instruments and methodologies without compromising their services. How can this dual mission be achieved? Research institutes and universities striving to maintain their competitiveness should cultivate an integrated and collaborative approach that—amongst others—embraces technology in all its aspects, from scouting to service.

## Scouting as a dedicated responsibility

It has become impossible for an individual scientist to master the influx of technology platforms that accelerate the speed of discovery. Although the symbiotic relationship between science and technology has always been a driver of progress, the pace and balance have shifted dramatically. Today, we are in the midst of an exponential technological leap and institutes all around the globe have to evolve and participate in this fundamental shift in modern science.

… we are in the midst of an exponential technological leap and institutes all around the globe have to evolve and participate in this fundamental shift in modern science.

That is why we believe a central scouting system is essential to stay ahead and master the arrival of new platform techniques, to separate the wheat from the chaff, and to grasp the opportunity offered by techniques with game-changing potential. At VIB, we launched a new way of working in 2008 with a dedicated “Tech Watch” team of tech-savvy scientists with the sole mandate to scout, testify, de-risk and benchmark platforms in beta-testing phase or even before. As a result, the implementation of new techniques changed from ad hoc to a more structured approach. Technology de-risking became a cooperative effort, with Tech Watch team members as sidekicks of Principal Investigators (PI) and core facilities, which has provided the institute with competitive advantages. During the past decade, life science institutes across Europe acknowledged VIB’s Tech Watch initiative as an exemplary case of technology risk mitigation and started to adapt this strategy to their specific environment.

## The traditional and ‘reverted’ approach to early adoption

**Figure 1:**
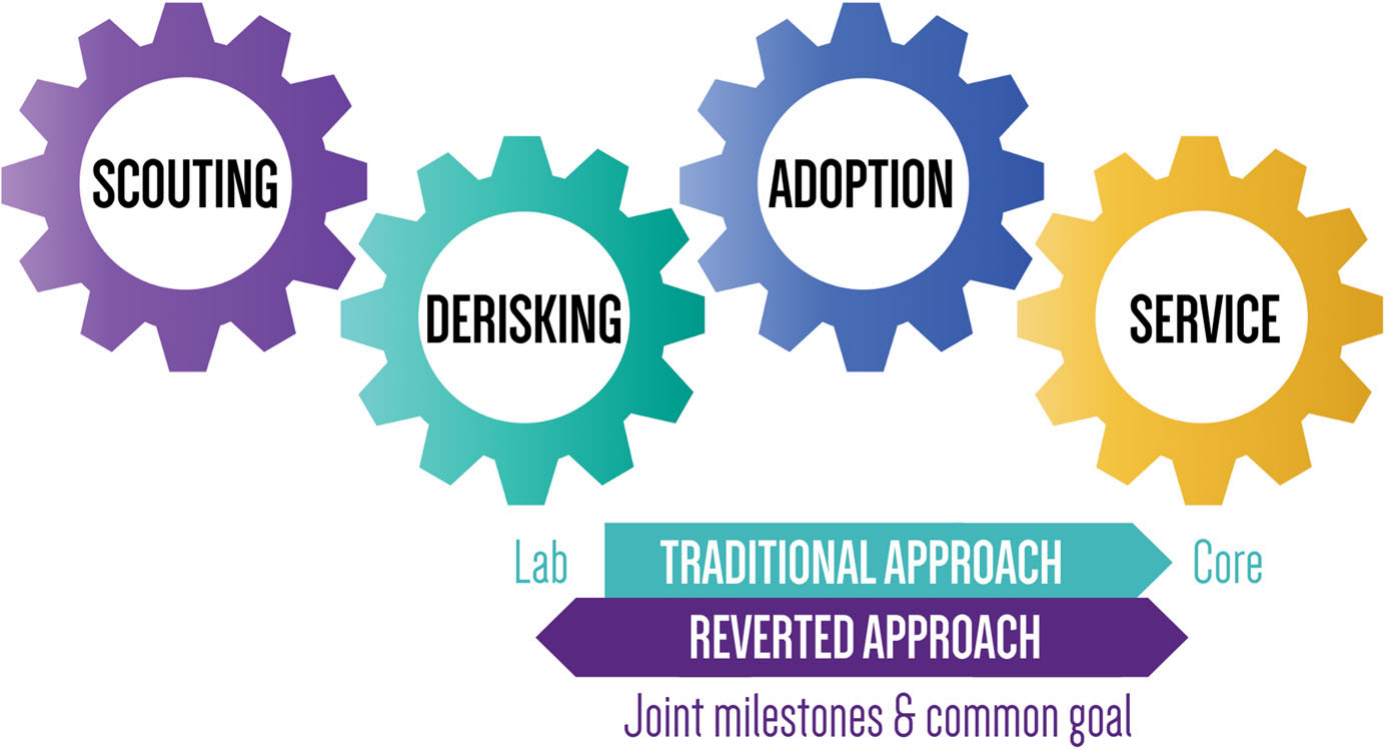
The traditional and ‘reverted’ approach to early adoption. Traditionally, new technologies are implemented within an individual research lab. When multiple labs want to apply the new method to different projects, at a certain point it becomes sensible to integrate the expertise into a support-focused unit or even a core facility to support institute-wide access. In what we call the ‘reverted approach’, a charter with incentives for both individual PIs and core facilities defines joint milestones so that new technology can be validated and, when deemed successful, made accessible proactively within the broader research community.

Traditionally, the introduction of a new technology usually follows a grassroots approach, whereby an individual PI implements a new method or technique within their own research lab. Subsequently, this innovation gains traction as other labs become interested in applying the new method to different projects. Over time, the technology generates sufficient traction beyond its original applications and reaches a critical point at which it becomes sensible to integrate this expertise into a support-focused unit that provides broader access. In cases where dissemination across the entire research organization is desired, this knowledge transfer eventually progresses to fully established core facilities with a mission to offer that service to both institute and external users. This ‘traditional’ approach has proven effective, but, with the rapid emergence and diversification of certain disciplines such as omics pipelines, single-cell and spatial technology, we realized that this way of operating can sometimes be too slow and inflexible to remain competitive (Fig. 1).

Often, research labs with early adopters adapt more swiftly to emerging fields and technologies than core facilities. Typically, their focus centers on a niche application specifically related to their biological research question. Expanding the potential of a technique beyond this scope requires labor-intensive sample-specific optimizations and additional de-risking, for which individual labs usually neither have the time and resources nor the incentive. From the perspective of a core facility, the confinement of techniques within individual labs is therefore a missed opportunity. Consequently, platform projects run the risk of failing to reach their full potential and can end up in a corner collecting dust when the PI has published the results and moves on to a new project. Unintentionally, such pilot experiments with novel platforms often end up in a ‘valley of death’ instead of broadening their potential and value.

… platform projects run the risk of failing to reach their full potential and can end up in a corner collecting dust when the PI has published the results and moves on to a new project.

To avoid this, we are exploring early adoption routes beyond the traditional trajectory that incorporate incentives for both PIs and core facilities. On a case-by-case basis, we set up collaborations between various stakeholders—PIs, researchers, cores, and technology scouters—during the project’s design phase to jointly define tangible criteria and milestones, including a plan to provide financial support to the leading PI for time and effort spent on optimizing the new method. This ‘reverted’ approach shares a common goal: validation of the technology and, when deemed successful, sharing of experience, data and protocols proactively with core facilities, which enables them to rapidly deploy this knowledge and make it accessible to the broader university or institutional community. At VIB, we deploy both the traditional and ‘reverted’ approach, as they each have their own merits and can complement each other in different situations and contexts. This approach relies on a culture of trust and a collaborative spirit.

## Dedicated funding and knowledge-sharing models

We have explored different strategies to strengthen the uptake and testing of novel platforms at an early stage in the tech cycle, while keeping the scientific questions of our researchers in mind. One such initiative was VIB’s Single Cell Accelerator, a temporary vehicle that played a vital role in the early implementation of single-cell technology across our institute and ultimately served as a launching pad for a dedicated Single Cell Core.

The Single Cell Accelerator made an instant impact when project calls for funding were launched in 2018. The Tech Watch scouting expertise and framework, from application and procedures to templates and review guidelines, could be recycled and, in less than 3 years, the majority of the researchers at our institute integrated single-cell workflows in their research programs. In 2021, the Single Cell Accelerator was phased out. Simultaneously, VIB started preparations to launch the Single Cell Core, which was officially inaugurated in January 2022 and to date offers a continuously growing portfolio of applications. The fast-track implementation of 10XGenomics Chromium platforms was unprecedented: nowhere else in Europe did the transfer from one single demonstration set-up to a park of more than 20 bench-top cyclers transpire so fast. The organic transfer of knowledge between Tech Watch, Single Cell Accelerator, and Single Cell Core was a key success factor for this achievement.

… when embracing and adopting technological and methodological innovations, core facilities must keep the bigger picture in mind, that is, their mission to provide services to the scientific community.

A more recent example is the rapid technological progress in the field of spatial biology. This highly dynamic field remains in a state of constant flux, and which platforms will eventually become standards—as Illumina did a decade ago for next-generation sequencing—is unpredictable. Deciding to test or not to test any of the highly promising leads is a significant challenge. Many efforts to test new technology platforms and mitigate risks are fragmented and conducted by individual research labs in specialized pilot experiments. Entrusting this to a service facility is often considered too risky, which prompted us to seek a middle ground. VIB introduced the Spatial Catalyst to connect the dots of scattered information and expertise within our institute, inform the research community, and disseminate information to facilitate a swift incorporation of spatial techniques.

With the universal uptake of (multi-)omics- and spatial resolution-driven research, life sciences institutes across the globe are realizing that their individual technology units have to complement their *modus operandi* with (virtually) integrated domain-spanning alternative formats. Agile, one-team efforts have been initiated at EMBL, ETH and CRG, and other leading institutes. Although the names of these new structures that facilitate low input- and resolution omics studies—often including organ-on-a-chip technology—vary from Hubs, User Labs to Integrated Project Teams, their intrinsic nature and mission are similar: rapid gathering and transfer of knowledge at the edges of technology-driven disciplines.

## Striking a balance between innovation and service

Our vision of technology adoption entails fostering a culture of change, not for the sake of it but to fulfill a scientific mission. However, such changes require careful planning to ensure innovation does not disrupt routine services. Timing is critical, because a ‘good’ decision that comes too early or too late may lead to a dead end. All of this means that, when embracing and adopting technological and methodological innovations, core facilities must keep the bigger picture in mind, that is, their mission to provide services to the scientific community.

This integration creates what we call a techno-scientific ecosystem in which core facilities must remain agile to embrace the latest advancements in scientific instruments and methodologies, without compromising core services. At VIB, we regularly review and adapt our portfolios and access models. We frequently question if it is opportune to fade-out particular branches, or scout for essential new applications and determine the best time and strategy to implement them. We actively monitor our surroundings and seek input from our researchers and partners, fully aware that staying static can harm our success. The skills and composition of the team need to accommodate this continuous balancing act between service and innovation.

## The techno-scientific realm is multidisciplinary by definition

The continuous pressure to invest in new cutting-edge technology at such a fast pace is typical for biology. Therefore, continuous innovation is per se a must and a challenge for the community. However, the complexity of the techno-scientific realm doesn’t stop there. Through the use of cutting-edge technology as a tool to map fundamental events underlying disease phenotypes, we have seen the growth of cross-disciplinary approaches, bridging distant fields like immunology and cancer, neurology, and systems biology. This collaborative, theme-expanding approach has yielded valuable insights, benefiting patients and society at large. A prime example is immunotherapy that is transforming cancer treatment.

During the past decade, it has also become clear that translational approaches are crucial in addition to mechanistic research. One doesn’t replace the other, but to successfully navigate the valorization process, a combination of both disciplines is often essential. Together with the basic research and technology axes, translational bench-to-bed work and *vice versa* forms a third axis of modern life sciences. Completing this inter-connected framework, the fourth axis is data as the common link between the other three domains.

Teaching the next generation of scientists the basics for each of the four axes—biology, technology, translational science, and data science—is crucial and should begin promptly.

**Figure 2:**
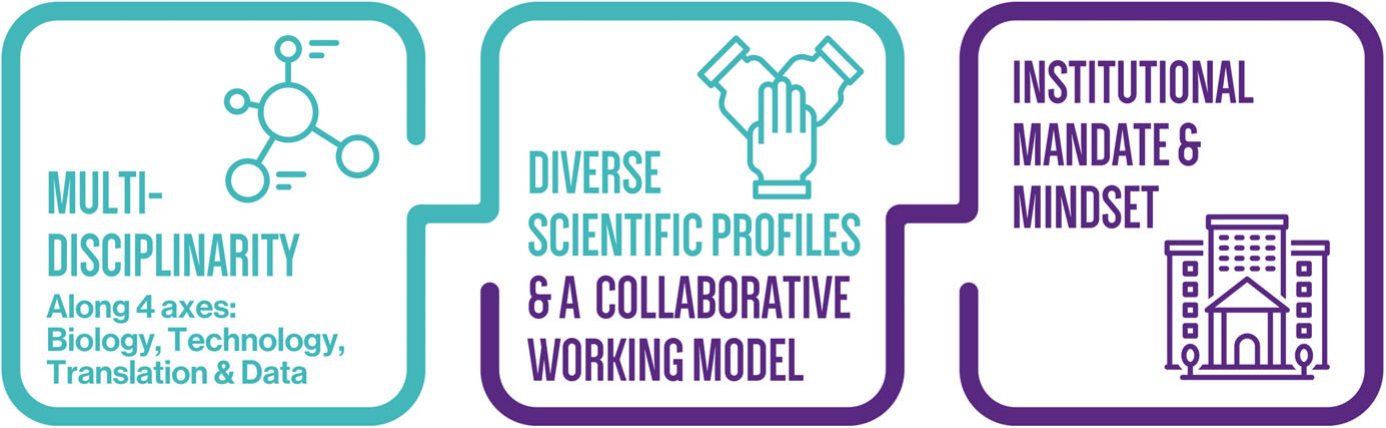
Building a future-proof techno-scientific environment. Successfully navigating the tech cycle requires embracing the multi-disciplinarity of life science research today, not only when it comes to the biology and technology itself, but also in relation to translational science and data science. By extension, it requires diverse scientific profiles with complementary expertise to that of traditional research academics (technology scouters and specialists, data scientists, business developers, and so on) through close and early collaboration. This can only work when the appropriate institutional mandate, mindset, and culture are in place.

Undoubtedly, as a community we are stepping into a new era of scientific research, which necessitates the education of future scientists with a strong emphasis on interdisciplinary skills. Teaching the next generation of scientists the basics for each of the four axes—biology, technology, translational science, and data science—is crucial and should begin promptly (Fig. 2). Moreover, continuous learning and training will remain an ongoing necessity throughout their careers, even more than it is today.

… only institutes where management fully integrates technology in their strategy roadmap will create an environment where impactful research can strive.

Those institutes that aim to conduct impactful research need to act at the intersection of disciplines, embrace technology in all its facets, and be fully prepared to handle the tsunami of increasingly complex data sets that will be generated through converging, integrated activities. The increased complexity has created a black-and-white scenario: only institutes where management fully integrates technology in their strategy roadmap will create an environment where impactful research can strive.

But such a joint technology mission does not materialize overnight. It takes years to establish a shared language, define a common mission, build trust, learn to collaborate across disciplines, create incentives for all involved parties, and, most importantly, demonstrate effectiveness through action. And while crafting the mandate is silver, translating it into practice is gold. Rome wasn’t built in a day, and preparing minds for collaborative techno-scientific work is a gradual process. Our advice? Begin tomorrow for it takes time before your tech enthusiasts can discern the ideal recipes for gathering and transferring knowledge at an optimal pace.

### Supplementary information

Peer Review File

