# Supplementary material for: Can you keep up? The challenges for research institutes and core facilities in scouting and adopting new technologies
Source: EMBO Rep. 2024 Feb 19;25(4):1704–7. doi: 10.1038/s44319-024-00078-w (PMC11014991; doi:10.1038/s44319-024-00078-w)
Supplement: Supplementary file 1 — Peer Review File [file 44319_2024_78_MOESM1_ESM.pdf]

EMBO reports

Can you keep up? The challenges for research institutes and core facilities in scouting and adopting new technologies

Geert Van Minnebruggen and Saskia Lippens
DOI: 10.15252/embr.202458841

Corresponding author(s): Geert Van Minnebruggen (geert.vanminnebruggen@vib.be)

**Review Timeline:**	Submission Date:	18th Jan 24
	Accepted:	24th Jan 24

Editor: Holger Breithaupt

Transaction Report: This manuscript was editorially reviewed.
